# Simvastatin Ameliorates Diabetic Cardiomyopathy by Attenuating Oxidative Stress and Inflammation in Rats

**DOI:** 10.1155/2017/1092015

**Published:** 2017-09-12

**Authors:** Nawal M. Al-Rasheed, Nouf M. Al-Rasheed, Iman H. Hasan, Maha A. Al-Amin, Hanaa N. Al-Ajmi, Raeesa A. Mohamad, Ayman M. Mahmoud

**Affiliations:** ^1^Department of Pharmacology and Toxicology, College of Pharmacy, King Saud University, Riyadh, Saudi Arabia; ^2^Department of Pharmaceutical Sciences, College of Pharmacy, Princess Nourah bint Abdulrahman University, Riyadh, Saudi Arabia; ^3^Department of Anatomy, College of Medicine, King Saud University, Riyadh, Saudi Arabia; ^4^Physiology Division, Department of Zoology, Faculty of Science, Beni-Suef University, Beni Suef, Egypt; ^5^Department of Endocrinology, Diabetes and Nutrition, Charité-University Medicine Berlin, Berlin, Germany; ^6^Department of Endocrinology, Diabetes and Nutrition at the Center for Cardiovascular Research (CCR), Charité-University Medicine Berlin, Berlin, Germany

## Abstract

Simvastatin is a lipid-lowering agent used to treat hypercholesterolemia and to reduce the risk of heart disease. This study scrutinized the beneficial effects of simvastatin on experimental diabetic cardiomyopathy (DCM), pointing to the role of hyperglycemia-induced oxidative stress and inflammation. Diabetes was induced by intraperitoneal injection of streptozotocin and both control and diabetic rats received simvastatin for 90 days. Diabetic rats showed significant cardiac hypertrophy, body weight loss, hyperglycemia, and hyperlipidemia. Serum creatine kinase MB (CK-MB) and troponin I showed a significant increase in diabetic rats. Simvastatin significantly improved body weight, attenuated hyperglycemia and hyperlipidemia, and ameliorated CK-MB and troponin I. Simvastatin prevented histological alterations and deposition of collagen in the heart of diabetic animals. Lipid peroxidation and nitric oxide were increased in the heart of diabetic rats whereas antioxidant defenses were decreased. These alterations were significantly reversed by simvastatin. In addition, simvastatin decreased serum inflammatory mediators and expression of NF-*κ*B in the diabetic heart. Cardiac caspase-3 was increased in the diabetic heart and decreased following treatment with simvastatin. In conclusion, our results suggest that simvastatin alleviates DCM by attenuating hyperglycemia/hyperlipidemia-induced oxidative stress, inflammation, and apoptosis.

## 1. Introduction

Cardiovascular disease (CVD) is the leading cause of morbidity and mortality in the world. Diabetes mellitus (DM) is a major contributing factor to CVD and heart failure [1]. A causative relationship between myocardial abnormalities and diabetes has been well demonstrated [2]. Diabetic cardiomyopathy (DCM) is the clinical condition associated with cardiac abnormalities provoked by diabetes [2]. It has been estimated that DCM affects approximately 12% of the diabetic patients and may lead to heart failure and death [3]. Cardiac hypertrophy, oxidative stress, inflammation, apoptosis, and myocardial interstitial fibrosis are the major features of DCM [4].

Persistent hyperglycemia in diabetes provokes excessive production of reactive oxygen species (ROS) and inflammation which play a key role in DCM [5, 6]. Hyperglycemia induces glucose auto-oxidation and surplus generation of ROS. Hyperlipidemia can also increase ROS production through stimulating nicotinamide adenine dinucleotide phosphate (NADPH) oxidases and inducing leakage of the mitochondrial electron transport chain [7]. Excess ROS activates protein kinase C and subsequently nuclear factor-*κ*B (NF-*κ*B), leading to myocardial injury [5, 6]. NF-*κ*B is a redox-sensitive protein complexes with a central role in inflammation [8]. Activated NF-*κ*B promotes the transcription and release of inflammatory mediators such as tumor necrosis factor-*α* (TNF-*α*) and thereby provokes myocardial inflammation [8].

Statins are hydroxymethylglutaryl coenzyme A (HMG-CoA) reductase inhibitors with lipid-lowering effect. Statins are the first-line treatment for coronary artery disease and are widely prescribed to prevent hypercholesterolemia [9]. Studies have shown the beneficial outcomes of statins on the cardiovascular system. In this context, Liu et al. [10] have recently reported that rosuvastatin postconditioning can protect isolated hearts against ischemia-reperfusion injury. Independently of their lipid-lowering effects, chronic pretreatment with statins preserved the integrity of microvasculature after acute myocardial infarction [11]. Improved myocardial perfusion and decreased infarction areas after ischemic reperfusion have been associated with early and chronic pretreatment with statins [11, 12]. The beneficial therapeutic outcomes of statins have been attributed to their ability to activate protein kinase B and endothelial nitric oxide synthase (eNOS) [13] and to attenuate oxidative stress [14, 15]. Previous studies have shown that simvastatin inhibits hypertrophy of cultured cardiomyocytes [16, 17] and in isoproterenol-induced rats [15]. In addition, simvastatin exerted protective effect against cardiac hypertrophy in a rat model of abdominal aortic constriction [18]. Recently, González-Herrera et al. [19] demonstrated that simvastatin can improve the pathophysiological condition in chronic Chagas cardiomyopathy experimental animal model. However, the protective effect of simvastatin against DCM has not yet been reported. Therefore, this study scrutinized the possible cardioprotective effect of simvastatin in a rat model of DCM.

## 2. Materials and Methods

### 2.1. Chemicals and Reagents

Simvastatin, streptozotocin (STZ), pyrogallol, reduced glutathione (GSH), malondialdehyde (MDA), thiobarbituric acid, and Griess reagent were supplied by Sigma-Aldrich (USA). Cholesterol, high-density lipoprotein (HDL) cholesterol, and triglyceride assay kits were supplied by Accurex (Mumbai, India). Antibodies for NF-*κ*B p65 (Cat. number sc-372), caspase-3 (Cat. number sc-7148), and *β*-actin (Cat. number sc-47778) were purchased from Santa Cruz Biotechnology (USA). Other chemicals and reagents were supplied by Sigma-Aldrich or other standard suppliers.

### 2.2. Experimental Animals

Ten-week old male Wistar rats weighing 160–180 g were included in this investigation. The animals were supplied by the College of Pharmacy at King Saud University (Riyadh, Saudi Arabia) and were provided free access to standard laboratory diet of known composition and water ad libitum. The rats were maintained at normal atmospheric temperature (23 ± 2°C) on a 12 h light/dark cycle. The experimental protocol and all animal procedures were approved by the Institutional Research Ethics Committee, College of Pharmacy at King Saud University (Riyadh, Saudi Arabia).

### 2.3. Experimental Design and Treatments

Type 1 DM was induced by a single intraperitoneal (i.p.) injection of 55 mg/kg body weight STZ to overnight fasted rats. STZ solution was freshly prepared by dissolving in 0.1 M cold citrate buffer (pH 4.5). Diabetes was confirmed through the determination of blood glucose levels at 72 hr using MEDISAFE MINI blood glucose reader (TERUMO Corporation, Tokyo, Japan). Rats with blood glucose levels higher than 200 mg/dl were considered diabetic and selected for further experiments. Diabetes was further verified by measuring blood glucose levels 7 days after STZ injection. Normal control rats received a single i.p. dose of physiological saline.

Thirty-two rats (16 diabetic and 16 normal) were divided into 4 groups (*N* = 8) as follows:

Group I (control): Nondiabetic rats received physiological saline orally for 90 days.

Group II (SIM): Nondiabetic rats received simvastatin (10 mg/kg) [15] dissolved in saline by oral gavage for 90 days.

Group III (diabetic): Diabetic rats received physiological saline by oral gavage for 90 days.

Group IV (diabetic + SIM): Diabetic rats received simvastatin (10 mg/kg) for 90 days.

### 2.4. Sample Collection and Preparation

Twenty-four hours after the last treatment, overnight fasted rats were sacrificed by cervical dislocation. Blood was collected and processed to separate serum. Hearts were excised, washed, and weighed. Samples from the heart were homogenized in cold phosphate-buffered saline (10% *w*/*v*), and clear homogenate was collected to assay MDA, nitric oxide (NO), GSH, and superoxide dismutase (SOD). Other samples were collected on neutral buffered formalin for histological and immunohistochemical processing while others were kept at −80°C for Western blotting.

### 2.5. Biochemical Assays

#### 2.5.1. Assay of Creatine Kinase MB (CK-MB) and Troponin I

Serum CK-MB and troponin I were determined using specific ELISA kits supplied by EIAab (Wuhan, China).

#### 2.5.2. Assay of Serum Lipids and Cardiovascular Risk Indices

Total cholesterol [20], triglycerides [21], and HDL cholesterol [22] were assayed in serum of normal and diabetic rats using reagent kits purchased from Accurex (Mumbai, India). Low-density lipoprotein (LDL) cholesterol level was determined using the formula

(1) $$\begin{array}{c} LDL\;cholesterol=total\;cholesterol-\left(\left[\frac{triglycerides}{5}\right]+HDL\;cholesterol\right). \end{array}$$

Very low-density lipoprotein (vLDL) cholesterol was calculated using the formula

(2) $$\begin{array}{c} vLDL\;cholesterol=\frac{triglycerides}{5}. \end{array}$$

Cardiovascular risk indices [23] and atherogenic index of plasma (AIP) were calculated as follows:

(3) $$\begin{array}{c} cardiovascular\;risk\;index\;1=\frac{total\;cholesterol}{HDL\;cholesterol},\\ cardiovascular\;risk\;index\;2=\frac{LDL\;cholesterol}{HDL\;cholesterol},\\ AIP={Log}_{10}\left(\frac{triglycerides}{HDL\;cholesterol}\right). \end{array}$$

#### 2.5.3. Assay of Tumor Necrosis Factor Alpha (TNF-*α*) and C-Reactive Protein (CRP)

Serum TNF-*α* and CRP were determined using specific rat ELISA kits purchased from Merck Millipore (USA) and Abcam (USA), respectively.

#### 2.5.4. Assay of Lipid Peroxidation, NO, GSH, and SOD

MDA, an index of lipid peroxidation, GSH, and SOD were assayed in the heart homogenates according to the methods described by Preuss et al. [24], Beutler et al. [25], Marklund and Marklund [26], respectively. NO in the heart homogenates was determined as nitrite content using Griess reagent [27].

### 2.6. Histopathology and Immunohistochemistry

Immediately after sacrifice, the hearts were excised, washed, and fixed in 10% neutral buffered formalin for 24 hr. Five *μ*m-thick paraffin sections were prepared, cut, and stained with hematoxylin and eosin (H&E). Other sections were stained with Masson's trichrome. Stained heart sections were scanned and examined using light microscopy.

Other heart sections were blocked via incubation in 3% hydrogen peroxide and washed in Tris-buffered saline (TBS; pH 7.6). The slides were incubated with protein block (Novocastra) to prevent nonspecific binding of antibodies and probed with rabbit anti-caspase-3 (Santa Cruz Biotechnology, USA). The sections were then probed with anti-rabbit secondary antibody, washed, and counterstained with hematoxylin. Negative control sections were similarly processed with omission of incubation with the primary antibody.

### 2.7. Western Blot

To investigate the effect of simvastatin on NF-*κ*B expression in the heart of normal and diabetic rats, Western blotting was applied as we recently reported [28]. Briefly, heart samples were homogenized in RIPA buffer containing proteinase inhibitors. Total protein content was assayed using Bradford reagent, and 40 *μ*g proteins were separated on SDS-PAGE, electrotransferred onto nitrocellulose membranes and blocked in 5% skimmed milk in TBS Tween 20. The blocked membranes were probed with rabbit anti-NF-*κ*B p65 and mouse anti-*β*-actin primary antibodies (Santa Cruz Biotechnology, USA). After washing, the membranes were incubated with the secondary antibodies and developed using enhanced chemiluminescence kit (Bio-Rad, USA). The blots were scanned and intensity of the obtained bands was quantified using ImageJ (NIH, USA). Results were normalized to *β*-actin and presented as percent of control.

### 2.8. Statistical Analysis

Results were presented as mean ± standard error of the mean (SEM). All statistical comparisons were made by means of the one-way ANOVA test followed by Tukey's test post hoc analysis using GraphPad Prism (GraphPad Software, CA, USA). A *P* value <0.05 was considered significant.

## 3. Results

### 3.1. Simvastatin Attenuates Body Weight Loss, Cardiac Hypertrophy, and Hyperglycemia in Diabetic Rats

**Figure 1:**
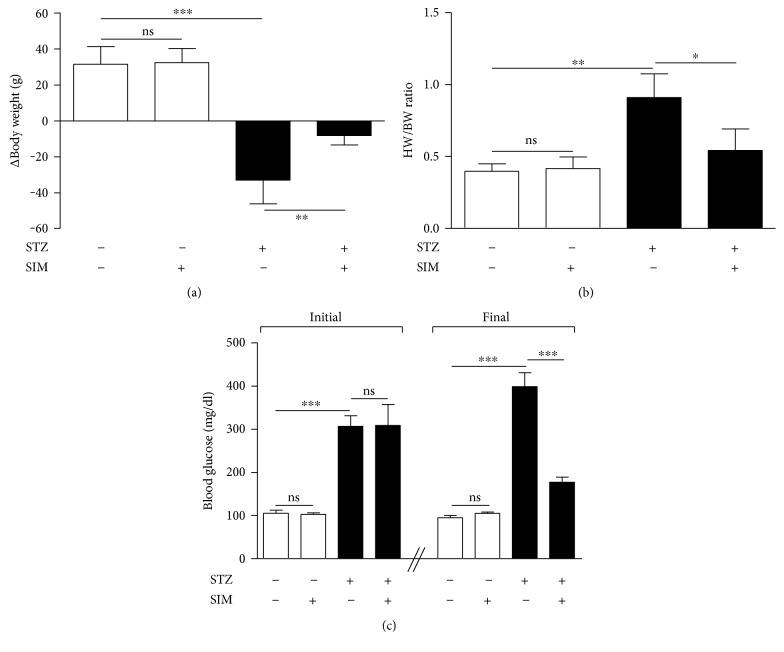
Simvastatin attenuates body weight loss (a), cardiac hypertrophy (b), and hyperglycemia (c) in STZ-induced diabetic rats. Data are *M* ± SEM (*N* = 8). ^∗^*P* < 05, ^∗∗^*P* < 0.01, and ^∗∗∗^*P* < 0.001. STZ: streptozotocin; SIM: simvastatin; HW: heart weight; BW: body weight; ns: nonsignificant.

Diabetic rats showed a significant (*P* < 0.001) body weight loss when compared with the control group (Figure 1(a)). Simvastatin significantly (*P* < 0.01) attenuated body weight loss in diabetic rats when supplemented for 90 days (Figure 1(a)). Normal rats that received simvastatin for 90 days showed nonsignificant (*P* > 0.05) changes in body weight when compared with the control group (Figure 1(a)).

Heart weight/body weight ratio (HW/BW) was significantly (*P* < 0.01) increased in diabetic rats when compared with the control group; an effect that was significantly (*P* < 0.05) repressed by simvastatin (Figure 1(b)). Simvastatin treatment for 90 days did not affect the HW/BW of normal rats as represented in Figure 1(b).

Blood glucose levels showed a significant (*P* < 0.001) increase when compared with the control rats (Figure 1(c)). Ninety days after, diabetic rats exhibited a significant (*P* < 0.001) increase in blood glucose when compared with the control rats. Simvastatin-treated diabetic rats showed a significant (*P* < 0.001) improvement in blood glucose levels when compared with the diabetic control rats (Figure 1(c)). Supplementation of simvastatin to normal rats did not affect blood glucose levels.

### 3.2. Simvastatin Prevents Hyperglycemia-Induced Damage and Collagen Deposition in the Diabetic Heart

To investigate hyperglycemia-induced myocardial injury and the possible protective role of simvastatin, we determined circulating CK-MB and troponin I levels and performed a histological study.

**Figure 2:**
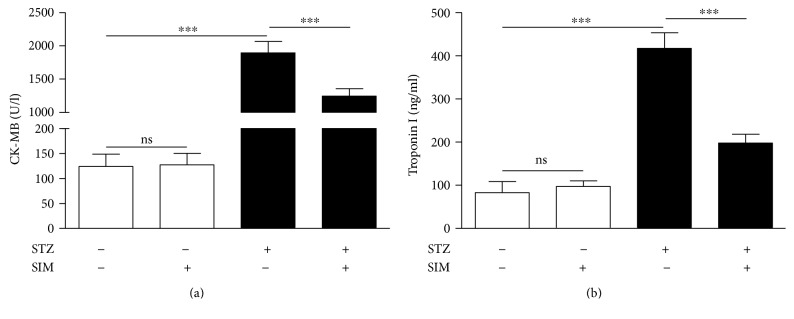
Simvastatin decreases serum CK-MB (a) and troponin I (b) levels in STZ-induced diabetic rats. Data are *M* ± SEM (*N* = 8). ^∗∗∗^*P* < 0.001. STZ: streptozotocin; SIM: simvastatin; CK-MB: creatine kinase MB; ns: nonsignificant.

Diabetic rats showed significantly (*P* < 0.001) increased serum CK-MB. In contrast, diabetic rats treated with simvastatin exhibited a marked (*P* < 0.001) improvement in CK-MB (Figure 2(a)).

Similarly, diabetic rats showed a notable (*P* < 0.001) elevation in circulating levels of troponin I as showed in Figure 2(b). Treatment with simvastatin markedly (*P* < 0.001) improved serum troponin I levels in diabetic rats.

Treatment of normal rats with simvastatin did not induce significant changes in either CK-MB or troponin I.

**Figure 3:**
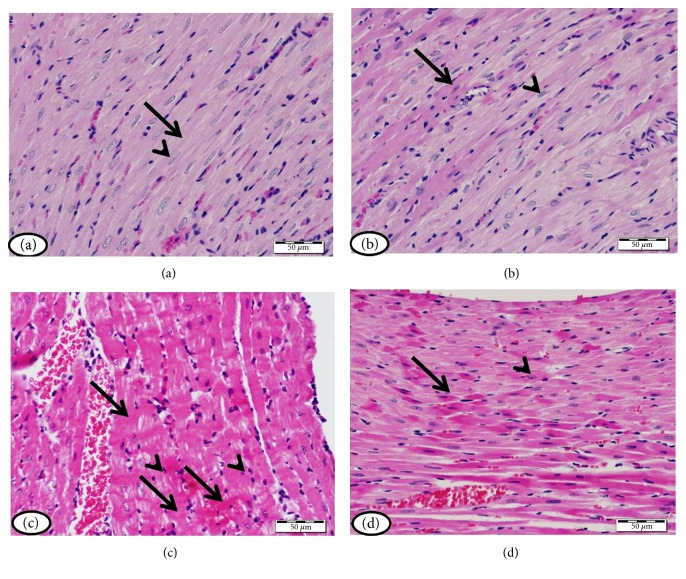
Heart of (a) normal control rats and (b) normal rats treated with simvastatin showing normal histological appearance of both myocardial cell cytoplasm (arrow) and nuclei (arrow head), (c) STZ-induced diabetic rats showing many myocardial cells with degenerated cytoplasm (arrow) and pyknotic nuclei (arrow head), and (d) diabetic rats treated with simvastatin showing decreased degeneration of myocardial cell cytoplasm (arrows) and nuclei (arrow heads). (H&E).

Histopathological study of heart sections of normal control rats revealed normal histological appearance of both cardiomyocytes cytoplasm and nuclei (Figure 3(a)). Normal rats treated with simvastatin exhibited normal heart histology as showed in Figure 3(b). On the contrary, heart sections of diabetic rats showed myocardial degeneration and pyknotic nuclei (Figure 3(c)). Simvastatin markedly prevented diabetes-induced myocardial damage as depicted in Figure 3(d).

**Figure 4:**
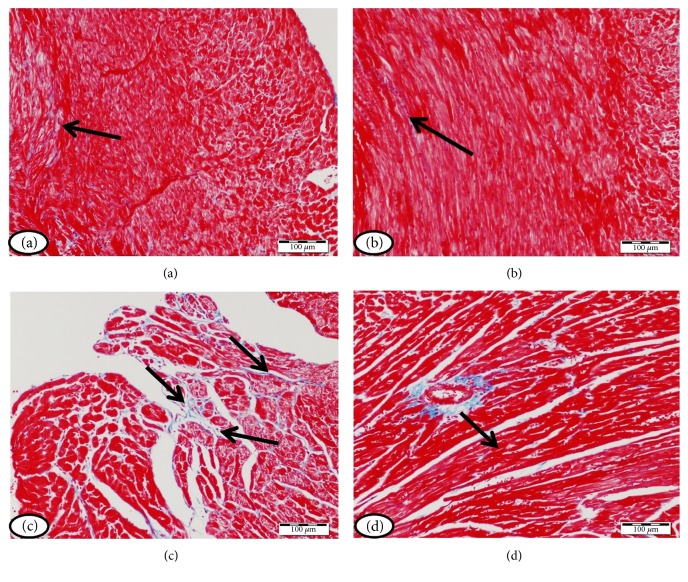
Heart of (a) control rats, (b) simvastatin-supplemented rats revealing normal amount and distribution of interstitial collagen (arrow), (c) STZ-induced diabetic rats with marked increase in the amount of collagen tissue in the endomysium especially surrounding blood vessels (arrows), and (d) diabetic rats treated with simvastatin showing decreased collagen deposition (arrow). (Masson's trichrome).

The heart sections showing collagen deposition were represented in Figure 4. Control (Figure 4(a)) and simvastatin-treated rats (Figure 4(b)) showed normal interstitial collagen. Diabetic rats showed increased collagen deposition in the endomysium, especially surrounding blood vessels (Figure 4(c)). Diabetes-associated collagen deposition was markedly prevented in the heart of diabetic rats treated with simvastatin (Figure 4(d)).

### 3.3. Simvastatin Ameliorates Hyperlipidemia and Prevents Atherogenesis in Diabetic Rats

**Figure 5:**
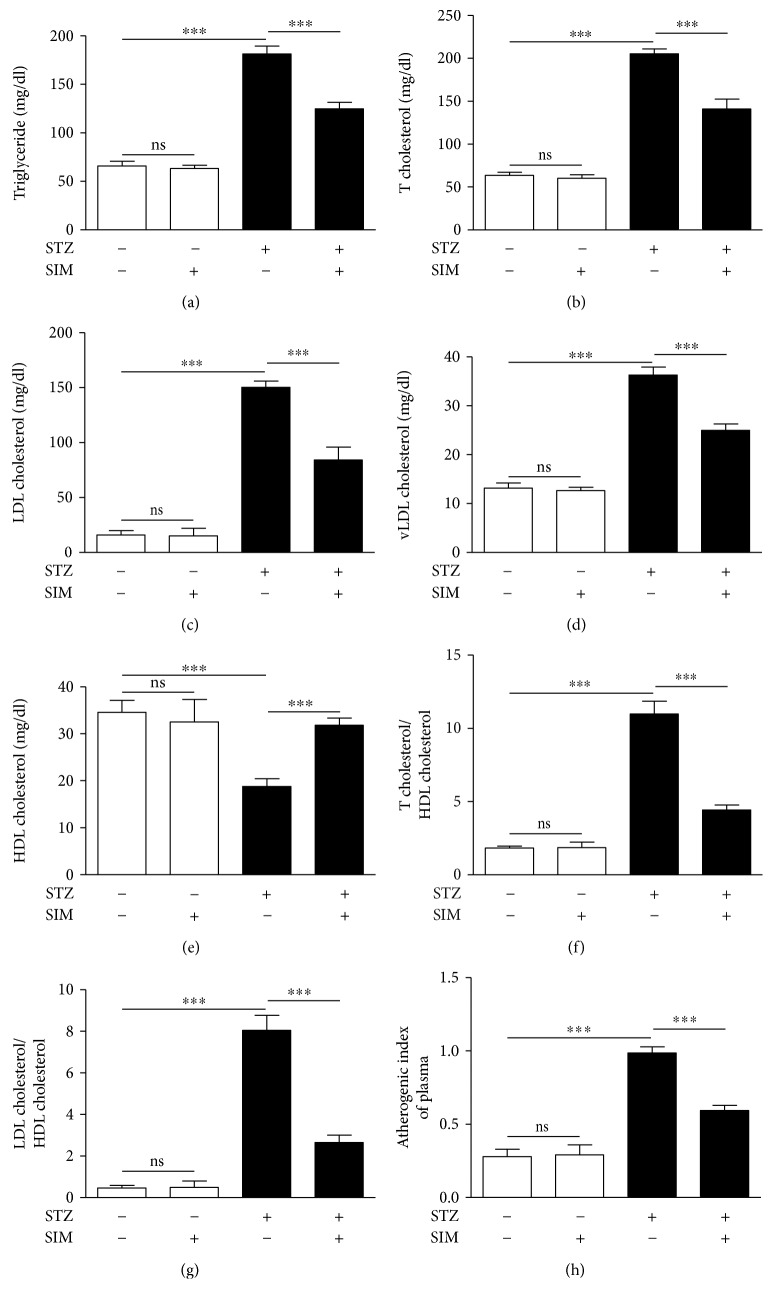
Simvastatin ameliorates hyperlipidemia and prevents atherogenesis in STZ-induced diabetic rats. Data are *M* ± SEM (*N* = 8). ^∗∗∗^*P* < 0.001. STZ: streptozotocin; SIM: simvastatin; T cholesterol: total cholesterol; LDL: low-density lipoprotein; HDL: high-density lipoprotein; vLDL: very low density lipoprotein; ns: nonsignificant.

The impact of simvastatin on serum lipids and cardiovascular risk indices is depicted in Figure 5.

Diabetic rats showed an atherogenic lipid profile characterized by significant (*P* < 0.001) increase in serum triglycerides (Figure 5(a)) and total (Figure 5(b)), LDL (Figure 5(c)), and vLDL cholesterol (Figure 5(d)) when compared with the normal control rats. HDL cholesterol was significantly (*P* < 0.001) declined in serum of STZ-induced diabetic rats when compared with the control group (Figure 5(e)). Treatment of the diabetic rats with simvastatin significantly (*P* < 0.001) reversed these lipid profile derangements.

To evaluate the impact of hyperlipidemia on the heart and blood vessels and the protective effect of simvastatin, the cardiovascular risk indices and AIP were determined. Cardiovascular risk indices showed a pronounced (*P* < 0.001) increase in STZ-induced diabetic rats (Figures 5(f) and 5(g)) and AIP (Figure 5(h)). Treatment with simvastatin significantly (*P* < 0.001) decreased cardiovascular risk indices and AIP of the diabetic rats.

Normal rats that received simvastatin for 90 days showed nonsignificant (*P* > 0.05) changes in lipid profile, cardiovascular risk indices, and AIP.

### 3.4. Simvastatin Suppresses Diabetes-Induced Oxidative Stress in the Diabetic Heart

The effect of simvastatin on the myocardial redox status of normal and diabetic rats was investigated via assessment of MDA, NO, GSH, and SOD.

**Figure 6:**
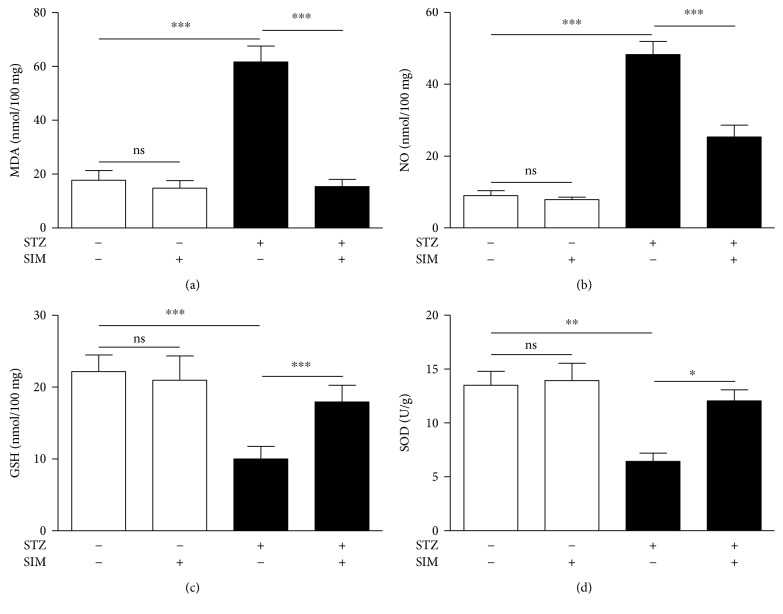
Simvastatin suppresses hyperglycemia-induced oxidative stress in the heart of STZ-induced diabetic rats. Data are *M* ± SEM (*N* = 8). ^∗^*P* < 0.05, ^∗∗^*P* < 0.01, and ^∗∗∗^*P* < 0.001. STZ: streptozotocin; SIM: simvastatin; MDA: malondialdehyde; NO: nitric oxide; GSH: reduced glutathione; SOD: superoxide dismutase; ns: nonsignificant.

Diabetic animals exhibited a remarkable (*P* < 0.001) increase in the myocardial MDA (Figure 6(a)) and NO (Figure 6(b)). Treatment with simvastatin significantly (*P* < 0.001) decreased cardiac levels of MDA and NO in diabetic rats. In normal rats, simvastatin administration for 90 days did not affect MDA and NO levels.

The antioxidant GSH was significantly (*P* < 0.001) declined in the heart of STZ-induced diabetic rats when compared with the control group (Figure 6(c)). In addition, diabetic rats showed a significant (*P* < 0.01) decrease in cardiac SOD activity (Figure 6(d)). Simvastatin, supplemented for 90 days, significantly ameliorated GSH content (*P* < 0.001) and SOD (*P* < 0.05) activity in the heart of diabetic rats while exerted nonsignificant (*P* > 0.05) effect in normal rats.

### 3.5. Simvastatin Inhibits Inflammation and Myocardial Apoptosis in Diabetic Rats

**Figure 7:**
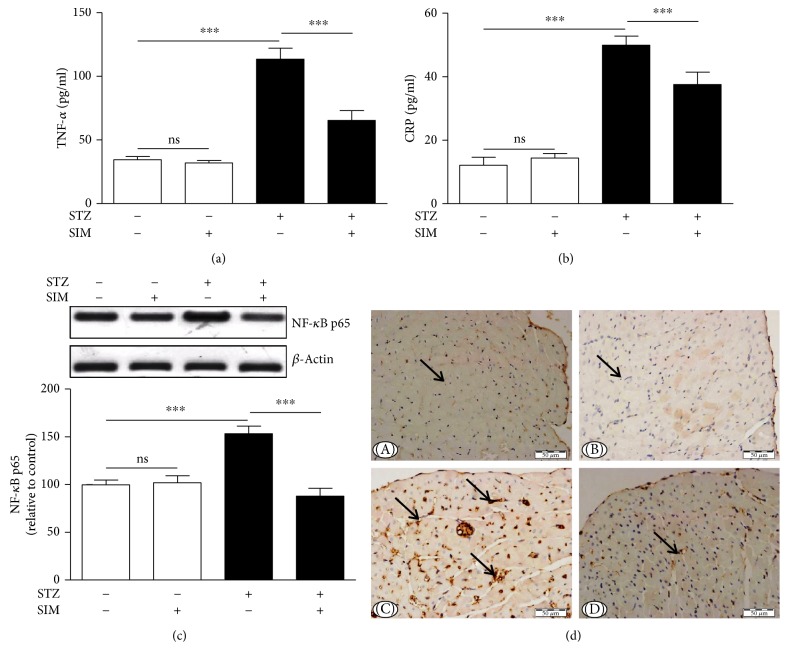
Simvastatin inhibits inflammation and myocardial apoptosis in STZ-induced diabetic rats. Simvastatin decreased serum levels of (a) TNF-*α* and (b) CRP and (c) NF-*κ*B p65 expression in the heart of diabetic rats. Data are *M* ± SEM (*N* = 8). ^∗∗∗^*P* < 0.001. STZ: streptozotocin; SIM: simvastatin; TNF-*α*: tumor necrosis factor alpha; CRP: C-reactive protein; NF-*κ*B: nuclear factor kappaB; ns: nonsignificant. (d) Photomicrographs of caspase-3 immunostained sections in the heart of (A) control rats, (B) normal rats treated with simvastatin showing immune-negative reaction (arrow), (C) STZ-induced diabetic rats showing strong immunopositive reaction in many myocardial cells (arrows), and (D) diabetic rats treated with simvastatin showing marked decrease in caspase-3 immune-positivity of the myocardial cells (arrow).

The ameliorative effect of simvastatin on diabetes-associated inflammation was explored via determination of serum TNF-*α* and CRP levels and cardiac NF-*κ*B expression. TNF-*α* (Figure 7(a)) and CRP (Figure 7(a)) were significantly (*P* < 0.001) increased in diabetic rats. Simvastatin significantly decreased TNF-*α* and CRP in serum of the STZ-induced diabetic rats. Oral administration of simvastatin to control rats exerted nonsignificant (*P* > 0.05) effects on serum TNF-*α* and CRP.

Protein expression of NF-*κ*B was significantly (*P* < 0.001) upregulated in the heart of STZ-induced diabetic rats (Figure 7(c)). Diabetic rats treated with simvastatin showed a significant (*P* < 0.001) downregulation of cardiac NF-*κ*B expression. Simvastatin did not affect NF-*κ*B expression levels in the heart of control rats (Figure 7(c)).

To investigate the protective effect of simvastatin on myocardial apoptosis in diabetes, the expression of caspase-3 was assessed by immunohistochemistry (Figure 7(d)). Heart sections of normal (Figure 7(d)-A) and simvastatin supplemented rats (Figure 7(d)-B) did not show caspase-3 immunopositive reaction of cardiomyocytes. STZ-induced diabetic rats showed marked increase in caspase-3 immunopositive stained cardiomyocytes (Figure 7(d)-C), an effect that was prevented by simvastatin (Figure 7(d)-D).

## 4. Discussion

Cardiomyopathy is an independent complication of DM which occurs in the absence of other heart diseases [29]. Hyperglycemia/hyperlipidemia-induced oxidative stress, inflammation, and apoptosis are probably involved in the pathogenesis of DCM [30, 31]. Here, we demonstrated the ameliorative potential of simvastatin on DCM in a rat model of STZ-induced diabetes. We provide the evidence that simvastatin ameliorates DCM via its ability to mitigate hyperglycemia, hyperlipidemia, oxidative stress, inflammation, and apoptosis.

The principal approach to control DM is lowering blood glucose levels. Here, STZ-induced diabetic rats exhibited a significant increase in blood glucose levels with concomitantly declined body weight as we previously reported [32]. Treatment with simvastatin significantly improved blood glucose levels in diabetic rats. Similar findings have been described in type 1 [14] and type 2 diabetic rodent models [33]. The antihyperglycemic effect of statins may be attributed to their pleotropic effects, including increased insulin release and improved insulin signaling and protection of pancreatic *β*-cells from ROS [34, 35]. Additionally, statins have been proven to inhibit the activity of dipeptidyl peptidase IV (DPP-IV) [36]. In the same context, we have recently reported attenuated hyperglycemia and DCM in diabetic rats treated with the DPP-IV inhibitor sitagliptin [32]. The improved glycemic status following simvastatin treatment may have contributed to the alleviated body weight.

Oxidative stress and inflammation are known to promote cardiomyocyte hypertrophy in hyperglycemic conditions [37]. Here, diabetic rats exhibited cardiac hypertrophy evidenced by the increased HW/BW ratio. Similar findings have been reported in our recent study [32]. In our study, cardiac hypertrophy was associated with increased TNF-*α* and cardiac NF-*κ*B expression. TNF-*α* has been reported to reduce degradation and increase synthesis of proteins in feline cardiomyocytes. These derangements occur through a mechanism involving preserved interaction between the extracellular matrix and cell integrins [38] and activation of NF-*κ*B [39]. Increased rate of fatty acid (FA) oxidation in the diabetic myocardium results in lipid accumulation and subsequently cardiac hypertrophy [40]. Myocardial hypertrophy has also been reported to occur after 8 weeks of diabetes [41]. Simvastatin treatment prevented cardiac hypertrophy in the diabetic rats. This effect might be attributed to the attenuated inflammation and oxidative stress. In consistent with our findings, simvastatin has prevented isoproterenol-induced cardiac hypertrophy in rats as we previously reported [15]. In addition, Liu et al. [18] showed the capacity of simvastatin to prevent *in vitro* and *in vivo* myocardial hypertrophy.

Hyperglycemia provoked cardiomyocyte damage as evidenced by the myocardium degeneration and pyknotic nuclei and elevated serum CK-MB and troponin I. These derangements are direct consequences of hyperglycemia-induced oxidative stress, inflammation, and other alterations. Elevated serum level of CK-MB is a powerful and sensitive tool to predict the risk of cardiac complications [42]. The onset of myofibrillar disintegration and inflammation-induced increase in permeability are associated with elevated circulating CK-MB and troponin I [43]. Previous research from our lab showed increased CK-MB and troponin I in serum of rat models of myocardial hypertrophy [15] and DCM [32]. Hyperlipidemia is another factor that contributes to cell death and cardiac dysfunctions in diabetes. Hyperlipidemia promotes the deposition of triglycerides and cholesterol in the myocardium and influences cardiac toxicological consequences [44]. Simvastatin significantly ameliorated indices of myocardial damage in diabetic animals. Therefore, reduced CK-MB and troponin I in simvastatin-treated diabetic rats supports the role of lipid-lowering agents in reducing hyperglycemia/hyperlipidemia-induced myocardial damage.

Given its role in inducing the production of ROS [45] and deposition of lipids in the myocardium [44], hyperlipidemia exerts a direct impact on the myocardium and increases the risk of coronary heart disease [46]. Here, STZ-induced diabetic rats exhibited an atherogenic lipid profile characterized by significant increases in serum cholesterol, triglycerides, LDL cholesterol, and vLDL cholesterol. In contrast, the cardioprotective HDL cholesterol [47] was significantly declined in the diabetic rats. Moreover, diabetic rats showed significantly increased values of the cardiovascular risk indices and AIP which is a frequently used predictor of atherosclerosis [48]. Simvastatin significantly reduced hyperlipidemia, cardiovascular risk indices, and atherogenic index in diabetic rats, indicating its potent lipid-lowering, antiatherogenic, and cardioprotective effects.

Counteracting oxidative stress is another mechanism we assumed have mediated the protective effect of simvastatin on DCM. Hyperglycemia/hyperlipidemia-induced production of ROS and reactive nitrogen species (RNS) constitutes a major contributing factor in the development of DCM. Hyperglycemia promotes generation of ROS and RNS in the mitochondria [49], and increased cardiac FAs activate NADPH oxidases and induce leakage of the mitochondrial electron transport chain [7]. Excess ROS promote upsurges in lipid peroxidation and consequently alter membrane structure and enzyme activity [50]. In the present study, diabetic rats exhibited significantly elevated lipid peroxidation and NO in the myocardium. NO and superoxide radicals can react and produce peroxynitrite, leading to DNA fragmentation and protein damage [51]. Diabetic rats exhibited reduced cardiac GSH, indicating its overutilization in the redox-challenged cellular microenvironment. GSH depletion induces further oxidative damage and necrotic cell death [52, 53]. SOD, an enzyme catalyzing the dismutation of superoxide, showed declined activity in the diabetic heart. Simvastatin reduced lipid peroxidation and NO and alleviated antioxidants in the diabetic heart. Accordingly, simvastatin decreased lipid peroxidation and increased hepatic and renal GSH levels in diabetic rats [14]. We have previously demonstrated the ability of simvastatin to decrease ROS generation and enhance antioxidant defenses in a rodent model of cardiac hypertrophy [15]. Therefore, the efficacy of simvastatin to attenuate oxidative stress may mediate, at least in part, its protective effect against DCM.

Cardiac inflammation, apoptosis, and fibrosis have been defined in the diabetic heart [31]. Seddon et al. [54] reported that cardiac inflammation occurs as a consequence of hyperglycemia-induced excessive ROS generation in diabetes. In our study, diabetic rats showed a significant increase in circulating TNF-*α* and CRP. Previous experimental studies demonstrated the contributing role of chronic inflammatory status in DCM [55–57]. TNF-*α* has been reported to exert a crucial role in the development of myocardial hypertrophy and dysfunction [55–57]. In the study of Bozkurt et al. [58], exogenous administration of TNF-*α* induced *in vivo* cardiac inflammation and dysfunction. The role of TNF-*α* in DCM has been further supported through observing similar effects in a mouse model with cardiomyocyte-specific TNF-*α* overexpression [59]. Additionally, in a murine model of pressure overload, genetic disruption of TNF-*α* hampered cardiac hypertrophy, dysfunction, and fibrosis [60]. In STZ-induced DCM, TNF-*α* antagonism protected against myocardial inflammation, leukocyte infiltration, and fibrosis [61].

Elevated CRP in STZ-induced diabetic rats in the present investigation was in agreement with the findings of previous studies showing increased serum CRP levels in diabetic patients [62] and STZ-induced type 1 diabetic animals [63]. Interestingly, diabetic rats treated with simvastatin showed significantly reduced serum TNF-*α* and CRP levels. Therefore, the beneficial effect of simvastatin is connected to its ability to reduce inflammation. In accordance, we have previously reported attenuated inflammation in a rat model of cardiac hypertrophy following simvastatin administration [15].

Next, we demonstrated the effect of simvastatin on NF-*κ*B expression in the diabetic heart. In consistent with the increased serum levels of TNF-*α* and CRP, cardiac NF-*κ*B was upregulated in the diabetic rats. Different mechanisms implicated in the establishment of inflammation in the diabetic myocardium were reported to converge towards NF-*κ*B activation. Hyperglycemia promotes NF-*κ*B transcription through activation of NADPH oxidase and production of ROS [64], degradation of I*κ*B [8], and activation of Erk1/2 [65] and mitogen-activated protein kinase (MAPK) [66]. Activated NF-*κ*B induces the upregulation of TNF-*α* and other molecules contributing to cardiovascular damage [8]. Increased circulating lipids may also contribute to NF-*κ*B activation in diabetes [67]. Treatment of the diabetic rats with simvastatin prevented NF-*κ*B activation in the myocardium, confirming its anti-inflammatory efficacy. In an experimental model of cardiac hypertrophy, we have reported the ability of simvastatin to reduce NF-*κ*B expression in the heart. This anti-inflammatory effect could be directly connected to its lipid-lowering mechanism.

Hyperglycemia-induced ROS generation can induce myocardial apoptosis and fibrosis [32, 54]. Myocardial inflammation promotes cardiomyocyte death and therefore contributes to cardiac remodeling [37]. Here, diabetic rats showed increased expression of the apoptosis marker caspase-3 in the myocardium. Apoptosis in the diabetic heart occurs as a direct result of sustained ROS production and inflammation. In this context, Haudek et al. [68] reported that sustained TNF-*α* signaling provokes both intrinsic and extrinsic cell death pathways, increases caspase-3 activation, and promotes cardiomyocyte apoptosis. The proapoptotic effects of TNF-*α* are likely mediated via NF-*κ*B activation [69]. Simvastatin markedly prevented hyperglycemia-induced cardiomyocyte apoptosis in STZ-induced diabetic rats via attenuating inflammation and ROS production.

Histological examination of the diabetic heart revealed myocardium degeneration and increased collagen deposition as we recently reported [32]. Oxidative stress, inflammation and cell injury are well-known causes of the cardiac fibrosis [61]. Cytokines and profibrotic factors released by cardiomyocytes and inflammatory cells stimulate fibrosis in the diabetic heart. Through its ability to activate WNT1 inducible-signaling pathway protein 1 (WISP1), TNF-*α* directly induces proliferation of cardiac fibroblast and production of collagen [70]. In addition, the profibrotic effects of cytokines have been shown to be boosted by ROS [55, 57]. Treatment with simvastatin markedly reduced collagen production and fibrosis, probably through its dual ability to enhance the antioxidant defenses and to reduce chronic inflammation in the heart of diabetic rats. Accordingly, statins have reduced cardiovascular events and mortality in diabetic patients [71]. Atorvastatin reduced myocardial inflammation and fibrosis in a diabetic rat model [72]. This effect was believed to be independent of atorvastatin's LDL cholesterol-lowering capacity [72]. Fluvastatin has also been reported to attenuate cardiac dysfunction and myocardial interstitial fibrosis in diabetes [73]. Therefore, lipid-lowering treatments seem to be effective in the attenuation of diabetes-associated cardiac fibrosis and cell injury together with a role in the primary prevention of the disease.

In conclusion, simvastatin has a protective effect on DCM. Simvastatin attenuated hyperglycemia/hyperlipidemia-induced oxidative stress and enhanced antioxidant defenses in the myocardium of diabetic rats. Simvastatin showed a strong modulatory effect against cardiac hypertrophy, inflammation, apoptosis, and fibrosis. Therefore, simvastatin and possibly other lipid-lowering agents can protect against DCM.
